# Supplementary material for: Health-Promoting Quality of Life at Work during the COVID-19 Pandemic: A 12-Month Longitudinal Study on the Work-Related Sense of Coherence in Acute Care Healthcare Professionals
Source: Int J Environ Res Public Health. 2022 May 16;19(10):6053. doi: 10.3390/ijerph19106053 (PMC9140864; doi:10.3390/ijerph19106053)
Supplement: Supplementary file 1 [file ijerph-19-06053-s001.zip › ijerph-1667150-supplementary.pdf]

Supplementary Materials

Table S1. Results of the two-level regression with time random effects for COVID-19–related anxiety of the healthcare professionals (n = 520; observations = 2368).

**Analysis**	**Variables**	**Model**					
		**1**		**2**		**3**	
		Unconditional cubic growth		Conditional cubic growth, 2-way cross-level interaction		Conditional cubic growth, 3-way cross-level interaction	
		b	**SE**	***b***	**SE**	***b***	**SE**
Fixed effects	Intercept	40.79***	0.20	39.42 ***	0.92	39.30***	0.93
Level I	Time	−1.05***	0.21	−1.45 ***	0.21	−1.037***	0.21
	$(Time)^3$	0.24	0.06	0.24 ***	0.06	0.25***	0.06
Level II	Female			0.28	0.30	0.27	0.30
	Age			0.00	0.02	0.00	0.01
	No-risk population			−0.29	0.44	−0.28	0.44
	No children			−0.44	0.36	−0.46	0.36
	Live alone			−0.26	0.48	−0.25	0.45
	Contact with risk population			1.91**	0.69	1.95**	0.69
	No relationship			0.02	0.45	0.02	0.45
	Infected during study			0.03	0.08	0.03	0.08
	Second-line HCP			−0.44	0.32	−0.26	0.41
	Workplace			0.00	0.09	0.00	0.09
	Sense of Coherence			−0.07***	0.02	−0.08***	0.03
Cross-level	$(Time)^3$*Sense of Coherence			0.01***	0.00	0.01	0.00
	$(Time)^3$*Second-line HCP					−0.02	0.04
	Second-line HCP*Sense of Coherence					0.03	0.04
	$(Time)^3$*Sense of Coherence*Second-line HCP					0.00	0.00
Variance components		**Estimate**		**Estimate**		**Estimate**	
Within participants (Level 1)		12.80		12.85		12.81	
Between participants (Level 2)		8.41		7.74		7.76	
Slope variance (Time)		6.41		5.85		5.81	
Slope variance $(Time)^3$		0.77		0.70		0.70	

* $p < 0.05$; ** $p < 0.01$; *** $p < 0.001$; HCP, healthcare professional; SE, standard error.

Table S2. Results of the multilevel analysis for perceived vulnerability to COVID-19 of the healthcare professionals (n = 520; observations = 2368).

**Analysis**	**Variables**	**Model**					
		**1**		**2**		**3**	
		Unconditional cubic growth		Conditional cubic growth, 2-way cross-level interaction		Conditional cubic growth, 3-way cross-level interaction	
		b	**SE**	***b***	**SE**	***b***	**SE**
Fixed effects	Intercept	54.62***	0.59	58.52***	2.88	58.55***	2.91
Level I	Time	0.21	0.51	0.20	0.51	0.20	0.51
	$(Time)^2$	−0.05	0.15	−0.05	0.15	−0.05	0.15
Level II	Female			1.51	0.95	1.52	0.95
	Age			−0.13*	0.05	−0.13*	0.05
	No-risk population			−3.02*	1.39	−3.03*	1.39
	No children			−1.94	1.11	−1.52	1.11
	Live alone			1.52	1.49	1.49	1.49
	Contact with risk population			−2.20	2.15	−2.25	2.15
	Relationship			−0.07	1.41	−0.04	1.41

	Infected during study			0.32	0.24	0.32	0.24
	Second-line HCP			1.14	0.98	1.13	1.26
	Workplace			−0.16	0.30	−0.16	0.30
	Sense of Coherence			−0.28**	0.06	−0.32***	0.08
Cross-level	(Time)^3*Sense of Coherence			0.01**	0.00	0.02***	0.01
	(Time)^3*Second-line HCP					0.00	0.09
	Second-line HCP*Sense of Coherence					0.09	0.12
	(Time)^3*Sense of Coherence*Second-line HCP						
Variance components		Estimate		Estimate		Estimate	
Within participants (Level 1)		58.85		58.63		58.62	
Between participants (Level 2)		129.18		119.28		119.66	
Slope variance (Time)		53.80		54.34		54.46	
Slope variance (Time)^3		6.60		6.55		6.55	

* $p < 0.05$; ** $p < 0.01$; *** $p < 0.001$; HCP, healthcare professional; SE, standard error; MarginalR2 0.72; 3-fach Interaktion $p = 0.097$.

Table S3. Results of the multilevel analysis for COVID-19–related depressiveness of the healthcare professionals (n = 520; observations = 2372).

**Analysis**	**Variables**	**Model**					
		**1**		**2**		**3**	
		Unconditional cubic growth		Conditional cubic growth, 2-way cross-level interaction		Conditional cubic growth, 3-way cross-level interaction	
		b	**SE**	***b***	**SE**	***b***	**SE**
Fixed effects	Intercept	5.50***	0.21	5.21***	0.93	5.44***	0.94
Level I	Time	−0.62***	0.19	−0.62***	0.19	−0.62***	0.19
	(Time)^3	0.16**	0.05	0.16**	0.05	0.12*	0.05
Level II	Female			1.05***	0.31	1.04***	0.31
	Age			−0.02	0.02	−0.02	0.02
	No-risk population			−1.30**	0.45	−1.30**	0.45
	No children			0.13	0.36	0.11	0.36
	Live alone			−0.05	0.48	−0.04	0.48
	Contact with risk population			−1.30	0.69	0.57	0.69
	Relationship			−0.21	0.46	−0.43	0.46
	Infected during study			0.21**	0.08	0.21**	0.08
	Second-line HCP			−0.10	0.32	−0.71	0.40
	Workplace			−0.04	0.10	−0.03	0.10
	Sense of Coherence			−0.19***	0.02	−0.19***	0.03
Cross-level	(Time)^2*Sense of Coherence			0.01***	0.00	0.01***	0.00
	(Time)^2*Second-line HCP					0.09**	0.04
	Second-line HCP*Sense of Coherence					0.02	0.04
	(Time)^2*Sense of Coherence*Second-line HCP					0.00	0.00
**Variance components**		**Estimate**		**Estimate**		**Estimate**	
Within participants (Level 1)		10.56		10.49		10.49	
Between participants (Level 2)		13.56		9.74		9.70	
Slope variance		3.65		3.82		3.79	
Slope variance (Time)^3		0.57		0.56		0.55	

*, $p < 0.05$; **, $p < 0.01$; ***, $p < 0.001$; HCP, healthcare professional; SE, standard error; Conditional R2 = 0.591; 3-fach interaction $p = 0.956$.

```
$contrasts
 contrast     estimate     SE  df t.ratio p.value
 0 - 9.43       -0.459 0.0661 503 -6.947  <.0001
 0 - -9.43       0.459 0.0661 503  6.947  <.0001
 9.43 - -9.43    0.918 0.1322 503  6.947  <.0001

Results are averaged over the levels of: Liner, Gender, Children, Househ
Degrees-of-freedom method: kenward-roger
P value adjustment: tukey method for comparing a family of 3 estimates
```

Table S4. Results of the multilevel analysis for COVID-19–related psychological trauma symptomatology of the healthcare professionals (n = 520; observations = 2371).

**Analysis**	**Variables**	**Model**					
		**1**		**2**		**3**	
		Unconditional cubic growth		Conditional cubic growth, 2-way cross-level interaction		Conditional cubic growth, 3-way cross-level interaction	
		b	**SE**	***b***	**SE**	***b***	**SE**
Fixed effects	Intercept	8.84***	0.22	8.26***	0.97	8.30***	0.98
Level I	Time	−1.49***	0.21	−1.48***	0.21	−1.48***	0.21
	(Time)^2	0.31***	0.06	0.31***	0.06	0.30***	0.06
Level II	Female			0.63*	0.32	0. 63	0.32
	Age			−0.04*	0.02	−0.04*	0.02
	No-risk population			−0.83	0.47	−0.82	0.47
	No children			−0.40	0.37	−0.42	0.38
	Live alone			0.36	0.50	0.37	0.50
	Contact with risk population			0.94	0.72	0.96	0.72
	Relationship			−0.64	0.48	−0.65	0.48
	Infected during study			0.19*	0.08	0.19*	0.08
	Second-line HCP			−0.20	0.33	−0.36	0.43
	Workplace			−0.01	0.10	−0.01	0.10
	Sense of Coherence			−0.19***	0.02	−0.20***	0.03
Cross-level	(Time)^3*Sense of Coherence			0.01***	0.00	0.01***	0.00
	(Time)^3*Second-line HCP					0.02	0.04
	Second-line HCP*Sense of Coherence					0.03	0.04
	(Time)^3*Sense of Coherence*Second-line HCP					0.00	0.00
**Variance components**		**Estimate**		**Estimate**		**Estimate**	
Within participants (Level 1)		13.47		13.45		13.44	
Between participants (Level 2)		12.61		9.66		9.70	
Slope variance (Time)		5.45		5.50		5.54	
Slope Variance (Time)^3		0.70		0.68		0.68	

*, $p < 0.05$; **, $p < 0.01$; ***, $p < 0.001$; HCP, healthcare professional; SE, standard error; Conditional R2: 0.543; 3-fach Interaktion $p = 0.757$.

```
$contrasts
 contrast     estimate     SE  df t.ratio p.value
 0 - 9.43       -0.466 0.0673 503 -6.929  <.0001
 0 - -9.43       0.466 0.0673 503  6.929  <.0001
 9.43 - -9.43    0.933 0.1346 503  6.929  <.0001

Results are averaged over the levels of: Liner, Gender, Children, Househol
Degrees-of-freedom method: kenward-roger
P value adjustment: tukey method for comparing a family of 3 estimates
```
